# Exploring the potential of the newly linked Growing up in England dataset for building up new evidence for Gypsy, Traveller and Roma young people.

**DOI:** 10.23889/ijpds.v7i3.2063

**Published:** 2022-08-25

**Authors:** Polina Obolenskaya

**Affiliations:** 1 London School of Economics

## Objectives

This paper aims to test and demonstrate the potential of the newly linked Growing up in England (GUIE) dataset for building up new evidence, knowledge and understanding of education and socio-economic circumstance of an often ‘invisible’ group of children and young people - Gypsy, Roma and Travellers.

## Approach

The paper utilises the first wave of the newly linked Growing up in England dataset, which consists of administrative education data for children and young people in schools and further education institutions in England linked to their 2011 Census records. It focuses on qualifications studied as well as attainment achieved of a cohorts of Gypsy, Traveller and Roma young people between age 15 (around the time of 2011 Census) and 19. The analysis explores the relationship between educational pathways and attainment for these young people and their household/family characteristics recorded in the 2011 Census.

## Results

This exploratory study shows that Gypsy, Traveller and Roma young people are more likely to be disadvantaged across multiple dimensions such as health and housing, and are less likely to achieve level 1, 2 and 3 educational qualifications compared to their peers.

## Conclusion

Evidence on outcomes for Gypsy, Traveller and Roma children and young people is scarce, in part due to limited available data on them. The newly linked GUIE dataset is therefore a rare opportunity to improve the evidence base for education and socio-economic outcomes for this group.

